# Overexpression of a Phosphate Starvation Response AP2/ERF Gene From Physic Nut in Arabidopsis Alters Root Morphological Traits and Phosphate Starvation-Induced Anthocyanin Accumulation

**DOI:** 10.3389/fpls.2018.01186

**Published:** 2018-08-20

**Authors:** Yanbo Chen, Pingzhi Wu, Qianqian Zhao, Yuehui Tang, Yaping Chen, Meiru Li, Huawu Jiang, Guojiang Wu

**Affiliations:** ^1^Key Laboratory of Plant Resources Conservation and Sustainable Utilization, South China Botanical Garden, Chinese Academy of Sciences, Guangzhou, China; ^2^College of Life Sciences, University of Chinese Academy of Sciences, Beijing, China

**Keywords:** AP2/ERF transcription factor, phosphate starvation, root morphology, anthocyanin, physic nut (*Jatropha curcas* L.)

## Abstract

Physic nut (*Jatropha curcas* L.) is highly tolerant of barren environments and a significant biofuel plant. To probe mechanisms of its tolerance mechanisms, we have analyzed genome-wide transcriptional profiles of 8-week-old physic nut seedlings subjected to Pi deficiency (P-) for 2 and 16 days, and Pi-sufficient conditions (P+) controls. We identified several phosphate transporters, purple acid phosphatases, and enzymes of membrane lipid metabolism among the 272 most differentially expressed genes. Genes of the miR399/PHO2 pathway (*IPS*, miR399, and members of the SPX family) showed alterations in expression. We also found that expression of several transcription factor genes was modulated by phosphate starvation stress in physic nut seedlings, including an AP2/ERF gene (*JcERF035*), which was down-regulated in both root and leaf tissues under Pi-deprivation. In *JcERF035*-overexpressing Arabidopsis lines both numbers and lengths of first-order lateral roots were dramatically reduced, but numbers of root hairs on the primary root tip were significantly elevated, under both P+ and P- conditions. Furthermore, the transgenic plants accumulated less anthocyanin but had similar Pi contents to wild-type plants under P-deficiency conditions. Expression levels of the tested genes related to anthocyanin biosynthesis and regulation, and genes induced by low phosphate, were significantly lower in shoots of transgenic lines than in wild-type plants under P-deficiency. Our data show that down-regulation of the *JcERF035* gene might contribute to the regulation of root system architecture and both biosynthesis and accumulation of anthocyanins in aerial tissues of plants under low Pi conditions.

## Introduction

Phosphorus (P) is an important macro-element for higher plants in the processes of growth and development, which is absorbed by the root system from soil in the form of inorganic phosphate (Pi) (Raghothama, 1999). In plants, Pi starvation stimulates changes in root system architecture (RSA), including inhibition of primary root growth accompanied by increases in lateral root number and both the length and number of root hairs (Péret et al., 2011). At the biochemical level, responses to Pi limitation include increases in production of phosphatases, RNases, Pi, H^+^ and organic acid transporters that facilitates Pi acquisition by roots (Vance et al., 2003; Plaxton and Tran, 2011). Another common plant response to phosphate deficiency is anthocyanin accumulation in the shoot (Marschner, 1995; Morcuende et al., 2007), which probably protects the photosynthetic apparatus and DNA from oxidative damage (Zeng et al., 2010). Comparative global gene expression profiling studies have shown that many Pi starvation response (PSR) genes are involved in these processes in Arabidopsis and other plants (Wasaki et al., 2003, 2006; Misson et al., 2005; Morcuende et al., 2007; Müller et al., 2007; Calderon-Vazquez et al., 2008; Li et al., 2010; Thibaud et al., 2010). These profiling studies indicate that Pi deprivation also triggers activation of alternative non-phosphorylating metabolic pathways. Moreover, during Pi deficiency, Pi is released from reserves contained in phospholipids, via their hydrolysis and conversion into sulfolipids or galactolipids (Misson et al., 2005).

Several studies have revealed important components of the sensing and signaling networks involved in Pi deficiency responses in Arabidopsis and rice. The MYB-CC transcription factors (TFs) PHOSPHATE STARVATION RESPONSE1 (PHR1) family genes in Arabidopsis (Rubio et al., 2001) and rice (Zhou et al., 2008; Guo et al., 2015) play key regulatory roles in these responses. A loss-of-function mutation in *PHR1* influences a subset of Pi starvation responses, including anthocyanin accumulation, changes in root to shoot growth ratios and expression of a subset of Pi starvation-induced genes (Rubio et al., 2001). SPX proteins play essential functions in regulating the activity of *AtPHR1*/*OsPHR2* under Pi starvation in distinct subcellular levels (Secco et al., 2012). In Arabidopsis, the miR399/PHO2 pathway operates downstream of *PHR1*, and regulates several Pi-dependent responses, such as Pi allocation between the shoot and root. Thus, the miR399/PHO2 pathway is a significant component of the Pi-signaling network (Lin S.I. et al., 2008; Chiou and Lin, 2011; Guo et al., 2015).

Besides the PHR1 family, members of the AP2/ERF (Ramaiah et al., 2014), bHLH (Yi et al., 2005; Chen et al., 2007), G2-like (Liu et al., 2009), R2R3 MYB and MYB-like (Devaiah et al., 2009; Dai et al., 2012; Yang et al., 2014; Nagarajan et al., 2016; Zhou et al., 2017), WRKY (Devaiah et al., 2007a; Chen et al., 2009; Wang et al., 2014; Su et al., 2015; Dai et al., 2016), and zinc finger (Devaiah et al., 2007b) TF families play essential roles in the regulation of Pi starvation responses. They affect several morphological processes that respond to Pi availability, and expression levels of several Pi starvation-induced (PSI) genes.

Physic nut (*Jatropha curcas* L.) is a species of shrubs of the family Euphorbiaceae. It can tolerate semiarid, drought-prone and barren environments, including low Pi environments that are not suitable for crop cultivation (Gubitz et al., 1999; Dhillon et al., 2009; Parthiban et al., 2009). Zhao et al. (2012) found that even after growing in a Pi-deficient medium for 17 days, physic nut seedlings can maintain high net photosynthetic rates, equivalent to ca. 90% of the rate of seedlings grown under Pi-sufficient conditions, despite significant declines in total Pi contents of their roots (ca. 55%) and aerial parts (ca. 81%). The dry weight of aerial parts decrease ca. 4%, whereas that of roots increase ca. 10%, compared with plants grown under Pi-sufficient condition (Zhao et al., 2012). However, the Pi starvation response mechanisms of physic nut, which control Pi homeostasis, remain obscure. Therefore, to probe these mechanisms we applied next-generation sequencing to explore transcriptomic changes in physic nut roots and leaves under Pi deficiency. Interestingly, we found that *JcERF035*, a member of the DREB subfamily of TFs, responded to Pi starvation in both roots and leaves. The DREB TFs play important roles in the responses to cold, dehydration, salt stress, and regulation of GA biosynthesis, cell dedifferentiation, plant morphology and branching (Lata and Prasad, 2011; Rehman and Mahmood, 2015). Previous studies indicate that DREB genes play important roles in determination of root architecture and abiotic stress responses (Liu et al., 2015; Liao et al., 2017; Yang et al., 2017). However, no DREB subfamily genes have been functionally characterized in terms of their responses to Pi deficiency in plants. Therefore, we overexpressed the *JcERF035* gene in Arabidopsis, and found that it altered root morphology, anthocyanin accumulation and expression levels of some PSI genes, but it did not significantly influence the Pi content of transgenic plants.

## Materials and Methods

### Plant Materials and Pi Starvation/Sufficiency Treatments

After disinfection with 1:5000 KMnO_4_ solution, seeds of the inbred physic nut cultivar GZQX0401 were planted in sand to germinate. When cotyledons were fully expanded, seedlings were transferred to trays containing a 3:1 mixture of sand and soil soaked with half-strength Hoagland solution in a greenhouse illuminated with natural sunlight. After emergence of the first true leaf, the trays were each irrigated with 1 L of Hoagland nutrient solution (pH 6.0) every 2 days at dusk. Pi deficiency (P-) and sufficiency (P+) treatments were begun at the six-leaf stage (8 weeks after germination). The P- treatment was initiated by removing most nutrients from the environments of a group of randomly selected seedlings by five washes, each with 1 L of tap water. These seedlings were then irrigated daily with Hoagland nutrient solution without phosphate, while another group, assigned to the P+ treatment, were not washed and irrigated with complete Hoagland solution (Zhao et al., 2012).

On the basis of our previous observation of changes in net photosynthetic rate (Pn) in physic nut leaves under Pi deficiency treatment, seedlings of the P- and P+ groups were sampled after 2 and 16 days of the treatment. According to these observations, Pn began to decrease during the first 2 days of the P- treatment, but remained at ca. 90% of the control rate after 16-day treatment (Zhao et al., 2012). The Pi content in Pi-deficiency group decreased 54.8 ± 3.5% and 81.2 ± 2.6% of controls’ contents in roots and shoots, respectively, while the root/shoot dry weight ratio increased from 0.047 ± 0.003 (control) to 0.054 ± 0.003 (Pi-deficiency) at the end of the treatment (17 days) (Zhao et al., 2012). In this study, roots and leaves of plants subjected to both the P- and P+ treatments were sampled at 8 a.m. to 9 a.m. after 2 and 16 days. Root samples consisted of 5–10 mm root tips, while leaf samples consisted of the second fully expanded leaf from the apex. Samples of both Pi-deficient and control groups were frozen immediately in liquid nitrogen and stored at -80°C. RNA isolation and digital gene expression library preparation and sequencing were performed as previously described (Zhang et al., 2014). For gene expression analysis, the numbers of expressed tags normalized to TPM (number of transcripts per million tags) were calculated (Zhang et al., 2014). We would prefer, ‘Data on the RNA-seq’ sequencing saturation of all samples are provided in **Supplementary Figure S1**.

### *JcERF035* Transformation

The full-length *JcERF035* coding domain sequence was cloned into the pCAMBIA1301 vector under control of the CaMV35S promoter (Tang et al., 2017). The resulting construct was transferred into Arabidopsis plants (Columbia ecotype) by the floral-dipping method (Clough and Bent, 1998). Homozygous lines with single T-DNA insertions were selected for subsequent analysis.

### Pi Deficiency/Sufficiency Treatments of Transgenic Arabidopsis Plants

Wild-type and transgenic Arabidopsis seeds were surface-sterilized and planted on half-strength Murashige and Skoog (MS) plates supplemented with 1% (w/v) sucrose and 1% (w/v) agar. The plates were placed at 4°C for 2 days in the dark, then positioned vertically in a growth chamber providing long-day photoperiod (16 h light/8 h dark) at 22 ± 2°C. Four days after germination (DAG), seedlings of all lines were transferred to new vertical Pi deficiency (P–) and Pi plus (P+) agar plates (Bates and Lynch, 1996), with the following macroelements: 3 mM KNO_3_, 2 mM Ca(NO_3_)_2_, 0.5 mM MgSO_4_, and 1 mM NH_4_H_2_PO_4_ (P+) or 0.5 mM (NH_4_)_2_SO_4_ (P-). After 7 days, the seedlings’ root morphology was observed and they were sampled for RNA isolation, anthocyanin assays, and quantification of total Pi.

### RNA Isolation, Semi-Quantitative RT-PCR, and Quantitative RT-PCR (qRT-PCR)

Total RNA was isolated from Arabidopsis leaves using Trizol^®^ reagent according to the manufacturer’s instructions (Invitrogen^1^), and from physic nut roots and leaves as previously described (Zhang et al., 2014). First-strand cDNAs were synthesized from 3 μg portions of total RNA, using M-MLV reverse transcriptase (Promega) following the manufacturer’s instructions. The primers used for RT-PCR in this work are listed in **Supplementary Table S1**. The *AtActin2* gene of Arabidopsis and *JcActin* gene of physic nut were used as references. Three independent biological replicates were performed for each PCR assay.

For semi-quantitative RT-PCR, PCR products were separated on 1.5% agarose gels and stained with ethidium bromide. The gels were then photographed using a Gel Imaging System (Shanghai Bio-Tech^2^), and an LCS480 system (Roche^3^) was used for quantitative real-time PCR. Each 20 μL reaction solution contained 10 μL 2 × SYBR Premix ExTaq, 0.4 μL forward primer (10 μM), 0.4 μL reverse primer (10 μM), 2 μL diluted cDNA solution, and 7.2 μL ddH_2_O. The thermal profile used for all PCR amplifications was: 10 min at 95°C for DNA polymerase activation, followed by 40 cycles of 5 s at 95°C, 20 s at 60°C and 20 s at 72°C. The 2^-ΔΔCT^ method was used for calculating the expression levels of genes.

### Measurements of Roots

A stereomicroscope (Leica M165C) equipped with a digital camera was used to capture images of root hairs within the zone 5 mm from the root tips of transgenic and wild-type seedlings at 4 DAG. The number and length of the root hairs of 30 seedlings from each line subjected to each treatment were measured using the ImageJ program^4^. The software was also used to record the primary root length, lateral root number, and lateral root length of 20 seedlings of each line subjected to each treatment.

### Anthocyanin Estimation

Anthocyanin contents of shoots of Arabidopsis plants were determined following Rabino and Mancinelli (1986). The anthocyanin was extracted from the shoots by incubating them in acidic (1% HCl, w/v) methanol at 4°C for 2 days. The absorbance (A) of the centrifuged extract was determined at 530 nm and 657 nm (A_530_ and A_657_, respectively), and the concentration of anthocyanin was expressed as A_530_-0.25A_657_ g^-1^ fresh weight.

### Quantification of Total Pi

Phosphate content was determined according to Ramaiah et al. (2014). Fresh samples ca. 50 mg were each transferred into a pre-weighed crucible and oven dried. After washing by furnace, samples were subsequently dissolved in 100 μL of concentrated hydrochloric acid. Then each sample was prepared by adding 10 μL the sample solution from the previous step and 790 μL ddH_2_O. A 200 μL portion of assay solution (35 mM ascorbic acid, 2.5 N H_2_SO_4_, and 4.8 mM NH_4_MoO_4_) was then added, and the resulting mixture was incubated at 45°C for 20 min. Appropriate standards were used to convert A_650_ values to total Pi contents, which were expressed as total Pi (nmol mg^-1^ dry weight).

### Statistical Analysis

Three to six biological repeats were used for all experiments, and the means acquired for all variables were compared using Duncan tests (Duncan, 1955) implemented in the SAS software package version 9^5^.

## Results

### Changes in Transcriptomic Profiles of Roots and Leaves of Physic Nut Seedlings in Response to Pi Starvation

For a preliminary screening of genes responsive to Pi-starvation in physic nut plants, roots and leaves sampled after 2 and 16 days of the P- and P+ treatments were used to examine genome-wide changes in the transcriptomes. Using the physic nut genome sequence we previously acquired (Wu P. et al., 2015), we identified transcripts with clean tags for a total of 272 protein-encoding genes that were severely affected (with ≥3-fold difference in expression level between Pi-deficient and Pi-sufficient plants) at all time points (**Supplementary Table S2**). About half of the up-regulated transcripts in leaves were orthologs of Arabidopsis genes that have been reportedly upregulated under Pi-starvation (**Supplementary Table S2**), including lipid metabolism, phosphate transporter, some C-metabolism, various phosphatase and SPX domain-containing genes (Misson et al., 2005; Müller et al., 2007). Many of the up-regulated genes were orthologs of Arabidopsis genes that are involved in soil Pi release, Pi uptake and Pi scavenging-related metabolism (**Supplementary Table S3**). The severely affected genes in Pi scavenging metabolic pathways were related to the glycolytic bypass, sulfate assimilate and reduction, fatty acid biosynthesis, and sulfo/galactolipid biosynthesis (**Supplementary Table S3**). Many of the severely down-regulated genes were involved in cell expansion, cell wall biosynthesis, and leaf extension (**Supplementary Table S2B**). Four AUX/IAA transcriptional regulator genes (JCGZ_01304, JCGZ_02271, JCGZ_07276, and JCGZ_23499) and two gibberellin-regulated family protein genes were down-regulated (JCGZ_19847 and JCGZ_25966).

**FIGURE 1:**
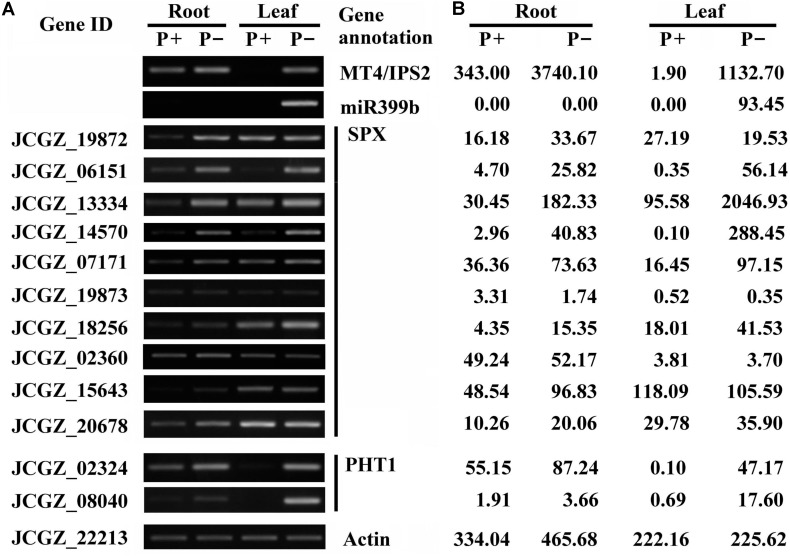
Results of RT-PCR analysis of differences in expression levels of selected genes involved in the miR399/PHO2 pathway and SPX family genes between physic nut seedlings grown under Pi deficiency (P–) and Pi sufficiency (P+). Root and leaf samples from plants 16 days after the onset of P– and P+ treatments were tested. **(A)** Results of semi-quantitative RT-PCR analysis showing expression of indicated genes relative to a physic nut *Actin* gene (bottom). **(B)** Expression changes obtained from the digital gene expression tag profiling database.

Several severely affected genes involved in the miR399/PHO2 pathway signaling pathway were also found. These included SPX domain-containing protein genes, miRNA399 precursor, ncRNAs of the IPS family, and *PHT1* transporter genes. Differential expression of ncRNAs, SPX domain-containing protein genes, and *PHT1* transporter genes in roots and shoots at 16 days after the onset of Pi deficiency was corroborated by RT-PCR and qRT-PCR (**Figure 1** and **Supplementary Figure S2**). According to the clean tags for these genes, no miRNA399 precursor was detected in roots and leaves under Pi-sufficient conditions, and there were low levels of transcripts for *MT4*/*IPS2*, *SPX* genes (JCGZ_06151 and JCGZ_14570), *PHT1* genes (JCGZ_08040 and JCGZ_02324) in Pi-sufficient leaves. In contrast, high levels of these transcripts were detected in Pi-deficient plants (**Figure 1**).

### Changes in Expression of *JcERF035* Under Pi Deficiency

**FIGURE 2:**
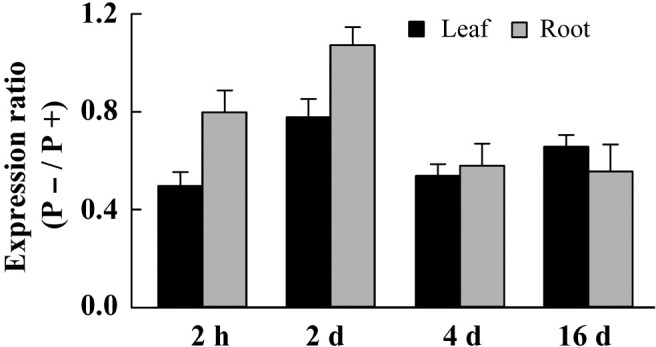
Differences in expression level of the *JcERF035* gene between leaves (black column) and roots (gray column) of physic nut seedlings grown under Pi deficiency (P–) and Pi sufficiency (P+). A physic nut *JcActin* gene was used as a loading control. Fold changes indicate ratios of expression levels under P– conditions to those recored under P+ conditions at each time point. Bars represent means ± SD from three biological replicates, each with two technical replicates.

Several TF genes responded to Pi starvation in physic nut, including genes of the WRKY (Xiong et al., 2013), NAC (Wu Z. et al., 2015), GRAS (Wu Z.Y. et al., 2015), MYB (Zhou et al., 2015), and AP2/ERF (Tang et al., 2016) families. We observed that an AP2/ERF gene, designated *JcERF035* (Accession No. JCGZ_24071 in GenBank), was down-regulated in both leaves and roots under the Pi deficiency treatment. The changes in expression of the *JcERF035* gene were confirmed by qRT-PCR. This revealed 22–51% reductions in the gene’s expression in leaves, and 20–44% reductions in roots, of seedlings after 2 h, 2, 4, and 16 days of the P- treatment, relative to those in Pi-sufficient seedlings (**Figure 2**).

### Overexpression of *JcERF035* in Arabidopsis Altered Root Morphological Traits

**FIGURE 3:**
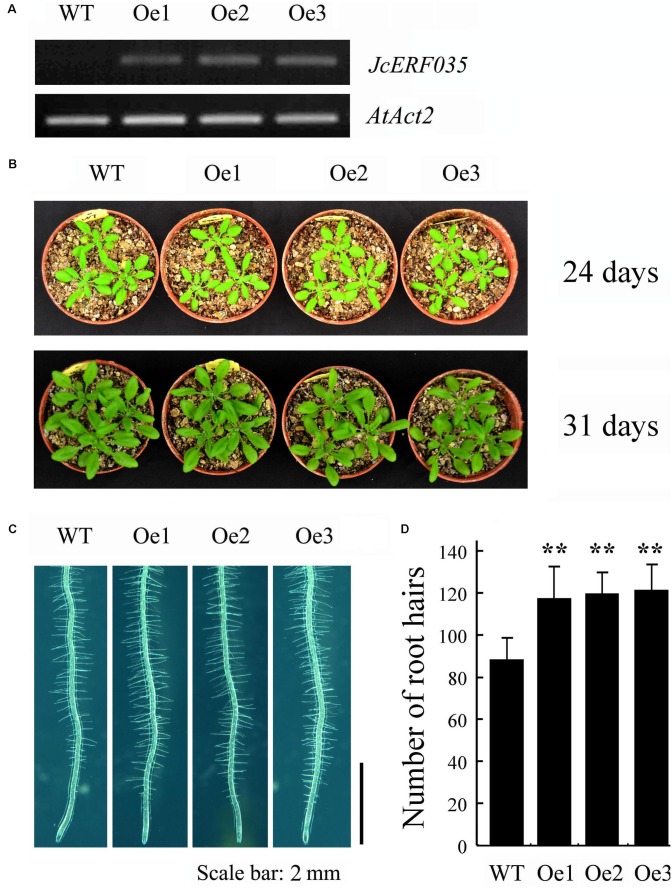
Overexpression of *JcERF035* (*OeJcERF035*) modulates root morphophysiological traits in Arabidopsis under normal conditions. **(A)** Relative expression levels of *JcERF035* transcripts in indicated transgenic lines (Oe1, Oe2, and Oe3) determined by semi-quantitative RT-PCR. **(B)** Plants 24 and 31 days old. **(C)** Phenotypes of root hairs in a 5-mm region of the primary root tip of 4-day-old wild-type and *OeJcERF035* lines grown on 1/2 MS plus 1% sucrose in agar plates. **(D)** Total numbers of root hairs in a 5-mm region of the primary root tip. Values shown are means ± SD from three biological replicates with 30 seedlings in each (Duncan test: ^∗∗^*P* < 0.01).

**FIGURE 4:**
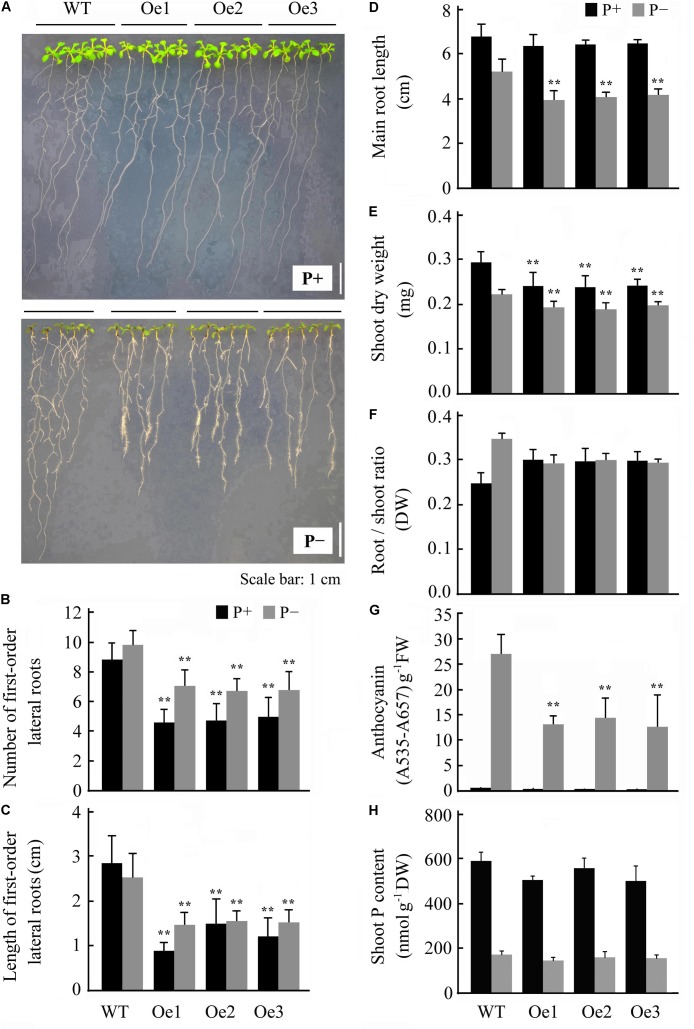
Overexpression of *JcERF035* modulates morphophysiological traits in Arabidopsis under Pi deficiency (P-) conditions at each time. Seedlings were germinated on 1/2 MS plus 1% sucrose medium for 4 days and then transferred to vertically oriented agar plates containing Pi-sufficient (P+) and P- media for 7 days. **(A)** Seedlings grown in P+ and P- agar plates. **(B,C)** Number **(B)** and total length **(C)** of first-order lateral roots. Values are means of *n* = 20 ± SD (Duncan test: ^∗∗^*P* < 0.01). **(D)** Primary root length. Values are means of *n* = 20 ± SD (Duncan test: ^∗∗^*P* < 0.01). **(E,F)** Dry weights of shoots **(E)** and root–shoot ratios **(F)**. Values are means of *n* = 20 ± SD (Duncan test: ^∗∗^*P* < 0.01). **(G,H)** Shoot anthocyanin contents **(G)** and Pi contents **(H)** of seedlings grown on agar plates. Values are means of *n* = 3 ± SD (Duncan test: ^∗∗^*P* < 0.01). Asterisks indicate significant differences between transgenic and wild type seedlings under the same growth conditions. DW, dry weight; FW, fresh weight.

Overexpression of *JcERF035* in Arabidopsis (*OeJcERF035*, or simply Oe lines hereafter) enhanced the sensitivity of transgenic seedlings to salt stress (**Supplementary Figure S3**), in accordance with reported results of overexpressing it in rice seedlings (Tang et al., 2017). To investigate functions of the physic nut *JcERF035* gene in response to Pi-deficiency *in planta*, three independent single insertion *OeJcERF035* lines (Oe1, Oe2, and Oe3) were used for detailed investigation. Semi-quantitative RT-PCR was used to detect the expression level of *JcERF035* in these transgenic Arabidopsis lines (**Figure 3A**). Apart from root morphology, no obvious developmental differences were detected between wild-type and transgenic lines under Pi-sufficient growth conditions on 1/2 MS medium in agar petri plates or vermiculite medium in pots (**Figures 3B**,**4A**). During growth in agar petri plates, we observed more root hairs in the basal 5-mm region of the primary root tip of *OeJcERF035* seedlings than in those of wild type plants (**Figures 3C,D**), accompanied by significant reductions in the number (**Figures 4A,B**) and total length (**Figure 4C**) of first-order lateral roots. Shoots of transgenic seedlings had ca. 20% lower dry weights than those of wild-type seedlings grown on vertically oriented agar plates (**Figures 4A,E**).

**FIGURE 5:**
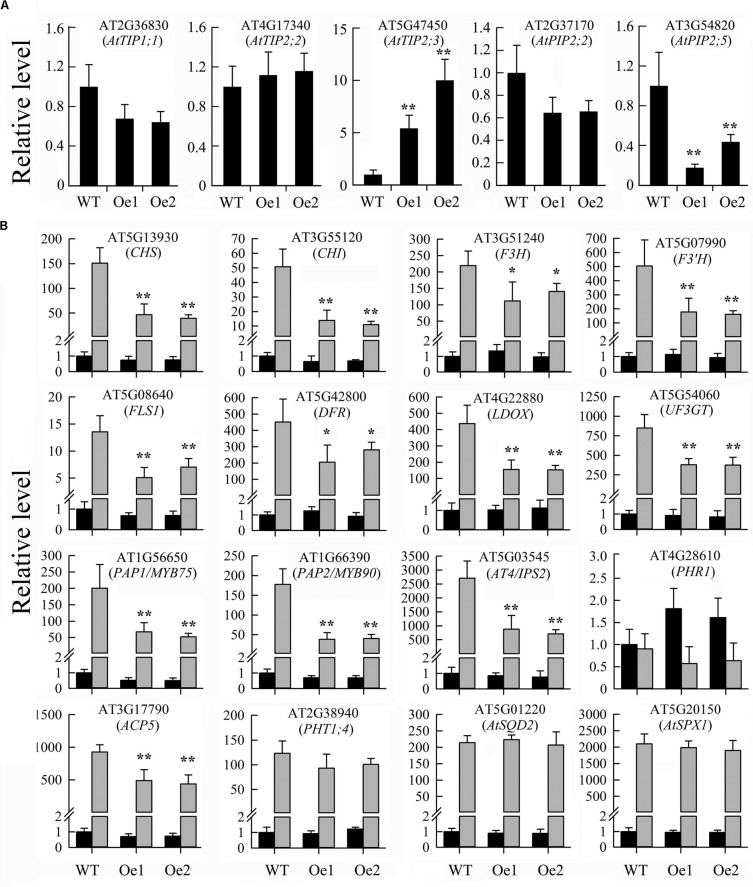
Results of quantitative real-time PCR analysis of gene expression levels in shoots of *OeJcERF035* seedlings: **(A)** Aquaporin genes, and **(B)** anthocyanin biosynthesis-related genes and some PSI genes. Wild-type (WT) and *JcERF035* overexpressing (Oe1 and Oe2) plants were germinated on half-strength MS medium for 5 d, then transferred to vertically oriented agar petri plates containing Pi-sufficient (P+) and Pi-deficient (P–) media for 7 days. Whole shoots were harvested and used for quantitative real-time PCR analysis to determine relative expression levels of genes in the wild type and transgenic lines. The Arabidopsis *AtActin2* (At3g18780) gene was used as a loading control. Expression values for all genes in wild-type plants under P+ conditions were set at 1. Values shown are means ± standard deviations from three biological replicates, each with two technical replicates. Asterisks indicate significant differences between transgenic and wild type seedlings e under the same growth conditions (Duncan test: ^∗^*P* < 0.05; ^∗∗^*P* < 0.01). Black columns = P+, gray columns = P–.

The *JcERF035* gene is a member of subgroup 1b of the ERF family and group A6 of the DREB subfamily (Tang et al., 2016). In Arabidopsis, several subgroup 1b ERF genes reportedly have roles in cell dedifferentiation and drought tolerance (Iwase et al., 2011; Zhu et al., 2014). In Arabidopsis lines overexpressing orthologs of *JcERF035* (At1g78080 or At1g36060), several aquaporin genes were found to be up-regulated more than twofold (Rae et al., 2011; Zhu et al., 2014). To determine whether these genes were also up-regulated in the *JcERF035*-overexpressing lines, we examined their expression levels in two *OeJcERF035* lines by qRT-PCR analysis. We found that the *AtTIP2;3* (At5g47450) gene was up-regulated more than twofold, whereas *AtPIP2;5* (At3g54820) was down-regulated more than twofold and the other three aquaporin genes—*AtPIP2;2* (At2g37170), *AtTIP1;1* (At2g36830), and *AtTIP2;2* (At4g17340)—showed less than twofold changes in expression in the *OeJcERF035* lines (**Figure 5**). Thus, the JcERF035 protein appears to regulate different genes from its Arabidopsis orthologs.

### Overexpression of *JcERF035* in Arabidopsis Affected Seedlings’ Responses to Phosphate Starvation

The *JcERF035* expression level was down-regulated in both roots and leaves of physic nut under Pi starvation stress. To examine the effect of this gene on phosphate starvation responses, 4-day-old wild-type and *OeJcERF035* Arabidopsis seedlings were transplanted into P- and P+ agar petri plates. *OeJcERF035* seedlings had fewer first-order lateral roots (**Figures 4A,B**), and lower shoot dry weights (**Figure 4E**) than the wild-type seedlings under both P+ conditions and P- conditions. Additionally, *OeJcERF035* seedlings had shorter primary roots and more root hairs on their primary root tips (**Figures 4A,D**), and lower root-shoot ratio (**Figure 4F**) under Pi deficiency.

The purple coloration induced by Pi starvation appeared lighter in *OeJcERF035* shoots than in wild-type shoots (**Figure 4A**), and the total anthocyanin content was notably lower in *OeJcERF035* shoots than in wild-type shoots under P- conditions (**Figure 4G**). To determine whether the lower anthocyanin content in *OeJcERF035* shoots was related to any effects of *JcERF035* overexpression on the maintenance of Pi homeostasis in the transgenic lines, we measured total Pi contents of shoots of the *OeJcERF035* and wild-type seedlings. Shoots of *OeJcERF035* seedlings had slightly lower Pi contents than the wild-type shoots under both P+ and P- conditions (**Figure 4H**). These results suggest that overexpression of *JcERF035* did not significantly modulate shoot Pi content in the transgenic seedlings.

### Changes in Expression of Phenylpropanoid Metabolism-Related Genes and Some PSI Genes in Shoots

To better understand the mechanisms underlying the observed reduction in phosphate starvation-induced anthocyanin biosynthesis conferred by overexpression of *JcERF035* in Arabidopsis, we tested the expression of several genes involved in phenylpropanoid metabolism and its regulation. We found that expression levels of most genes tested were significantly lower in *OeJcERF035* shoots than in wild-type shoots under Pi starvation conditions (**Figure 5**). These results suggest that the *JcERF035* gene is involved in suppression of Pi starvation-mediated anthocyanin biosynthesis in Arabidopsis.

Next, we analyzed expression levels of several PSI genes. Expression levels of phosphate transporter 1;4 (*PHT1;4*, At2g38940), *PHR1* (At4g28610), sulfoquinovosyldiacylglycerol 2 (*AtSQD2*, At5g01220), and *ATSPX1* (At5g20150) genes were not significantly affected. However, expression levels of *IPS2*/*AT4* (At5g03545) and purple acid phosphatase 17 (*ATPAP17*/*ATACP5*, At3g17790) genes were significantly lower in *OeJcERF035* shoots than in wild-type shoots under Pi starvation conditions (**Figure 5**). These results indicate that the *JcERF035* gene influences the expression of some, but not all, PSI genes in Arabidopsis.

## Discussion

Results of this study reveal molecular-level responses of physic nut roots and leaves to Pi deficiency. As reported in rice and Arabidopsis, many of the affected genes play roles in general metabolism. In roots, genes related to Pi release from soil, Pi uptake and transportation, lipid metabolism and the glycolytic bypass were most affected by Pi deficiency. In leaves, genes involved in membrane lipid and fatty acid metabolic pathways and the glycolytic bypass were most affected. In addition, genes involved in Pi transport and phosphatase-encoding genes were affected by Pi deficiency in both roots and leaves. Moreover, many genes involved in cell wall synthesis, cell wall extension and cell expansion were severely down-regulated in physic nut leaves by Pi deficiency. This is consistent with observed reductions in the size of plants’ shoots under Pi deficiency. The responses in expression of the *IPS*, miR399, and SPX family genes to Pi deficiency in physic nut were similar to those observed in rice and Arabidopsis (Bari et al., 2006; Secco et al., 2012), suggesting that the miR399/PHO2 inorganic phosphate homeostasis control network is also present in the woody plant physic nut. In Arabidopsis and rice leaves, genes involved in photosynthesis and general carbon metabolism are strongly influenced by Pi deficiency (Wasaki et al., 2006; Müller et al., 2007; Morcuende et al., 2007). We found that few photosynthesis and general carbon metabolism genes were severely influenced by low Pi in physic nut leaves, suggesting that physic nut is less sensitive to Pi starvation than rice and Arabidopsis.

Several differentially regulated genes encoding TFs that may contribute to transcriptional regulation of other genes under low Pi stress have been reported in Arabidopsis, rice, and other plants. In addition, several functionally characterized TFs have shown to be distinguishingly regulated under different Pi concentrations. They were involved in a number of morphological processes responding to Pi availability and in regulating the expression levels of some Pi starvation induced (PSI) genes. In this study, we found that the expression level of *JcERF035* was down-regulated under Pi deficiency (**Figure 2**), indicating that this gene may play a role in Pi nutrition stress responses. Overexpression of *JcERF035* did not seriously inhibit the growth and development of Arabidopsis plants under P+ conditions. Although the *JcERF035*-overexpressing lines displayed some reductions in number and length of lateral roots and shoot biomass (ca. 20%), their primary root lengths, flowering times, and inflorescence lengths were similar to those of wild-type plants. In sharp contrast, in *AtERF070* and *AtMYB62* overexpression lines the primary root length is also reduced and growth is seriously affected according to Devaiah et al. (2009) and Ramaiah et al. (2014).

To decipher the role of *JcERF035* in regulation of Pi starvation responses, three independent *OeJcERF035* Arabidopsis lines were analyzed. Appropriate alterations in root architecture are critical for plant to efficiently absorb and utilize the Pi in soil. The reductions in primary root length, proliferation of both lateral roots and root hairs close to the root apical meristem are typical responses of wild type Arabidopsis plants to Pi starvation (Williamson et al., 2001; Linkohr et al., 2002). The *OeJcERF035* Arabidopsis lines displayed reductions in primary root length, first-order lateral root number and length. Nevertheless, shoots of the *OeJcERF035* lines had similar Pi contents to those of wild-type plants under both P+ and P- conditions, possibly because *OeJcERF035* seedlings had more root hairs than the wild-type seedlings.

Root systems of diverse plant species are more branched and have higher root/shoot ratios under Pi-deficiency than under Pi-sufficiency. These changes in root architecture help to increase root systems’ capacities for soil exploration. We found that physic nut plants under P- conditions had 3.78 ± 0.08% lower shoot dry weights than P+ controls, but 9.95 ± 0.21% higher root dry weights. Thus, *JcERF035* may suppress lateral root development, and its down-regulation may promote increases in root system of physic nut plants grown under low Pi conditions, thereby enhancing their soil exploration capacity.

Although the *OeJcERF035* shoots had similar Pi contents to the wild-type shoots, they exhibited less anthocyanin accumulation under P- conditions. Expression levels of genes related to anthocyanin biosynthesis and its regulation, and some PSI genes, were also lower in *OeJcERF035* shoots than in wild-type shoots under P- conditions. These results imply that *JcERF035* might affect these processes independently of Pi content. Pi content-independent anthocyanin accumulation has also been observed in the *bhlh32* mutant of Arabidopsis, which has elevated Pi and anthocyanin contents under Pi-sufficient conditions (Chen et al., 2007). Genes involved in flavonoid and anthocyanin biosynthesis are up-regulated in aerial tissues of P-deficient plants (Hammond et al., 2003; Uhde-Stone et al., 2003; Wu et al., 2003). The accumulation of anthocyanins in the aerial tissues is a characteristic response of P-deficient plants, which is thought to protect nucleic acids from UV damage and chloroplasts from photoinhibitory damage caused by P-limited photosynthesis (Hoch et al., 2001). The down-regulation of the *JcERF035* gene in physic nut leaves might contribute to the biosynthesis and accumulation of flavonoids and anthocyanins in aerial tissues of plants grown under low Pi conditions.

The *JcERF035* gene is a member of subgroup 1b of the ERF family (group A6 of the DREB subfamily) (Tang et al., 2016). Arabidopsis has eight genes (known as *RAP2.4* genes) assigned to subgroup 1b: ATWIND 1–4, At1g64380, At2g22200, At4g13620, and At4g39780 (Nakano et al., 2006). Products of the *RAP2.4* genes participate in cell dedifferentiation, water balance and oxidative stress responses (Lin R.C. et al., 2008; Iwase et al., 2011; Rae et al., 2011; Zhu et al., 2014; Rudnik et al., 2017). Constitutive overexpression of At1g78080 causes defects in numerous developmental processes regulated by light and ethylene, including root elongation and root hair formation (Lin R.C. et al., 2008). Our previous work revealed that expression of *JcERF035* was down-regulated in physic nut leaves under salinity stress, and its expression in rice reduced expression of GA biosynthesis genes but increased the transgenic seedlings’ sensitivity to salt stress (Tang et al., 2017). Similar to the phenotype of overexpressing *JcERF035* rice seedlings, *OeJcERF035* enhanced the sensitivity of transgenic seedlings to the salt stress (**Supplementary Figure S3**). The *OeJcERF035* lines did not show any greater drought tolerance than wild-type controls (data not shown), nor any increase in expression levels of most aquaporin genes. In contrast, overexpression of WIND genes can increase expression levels of most aquaporin genes (Rae et al., 2011; Zhu et al., 2014). These results suggest that the *JcERF035* gene product functions differently from ATWIND in plants. Whether the WIND genes play roles in anthocyanin accumulation in aerial tissues under phosphate starvation conditions in Arabidopsis remains to be studied.

## Conclusion

The presented study has identified genes with diverse functions that appear to play important roles in adaptations of physic nut to Pi deficiency. The data obtained in this study expand available information on the regulatory and signaling pathways involved in Pi deficiency responses of plants, and should assist elucidation of the molecular basis of adaptation of plants to this stress. The *JcERF035* gene suppresses lateral root formation and phosphate starvation-induced anthocyanin accumulation in leaves. Thus, its down-regulation may promote increases in the root system and anthocyanin accumulation in aerial tissues of physic nut plants under phosphate starvation conditions. Our results indicated that a variety of adaptive changes help plants to cope with Pi deficiency, including induction of genes involved in positive regulatory responses and suppression of genes involved in physiological processes that must be inhibited to avoid Pi depletion.

## Author Contributions

The research was designed by GW, HJ, YpC, and ML. The experiments were performed by YbC, PW, QZ, and YT, and the data were analyzed by YbC and PW. The manuscript was written by YbC.

## Conflict of Interest Statement

The authors declare that the research was conducted in the absence of any commercial or financial relationships that could be construed as a potential conflict of interest. The reviewer LC declared a shared affiliation, though no other collaboration, with several of the authors YbC, QZ, and YT to the handling Editor.

## Footnotes

1 http://www.thermofisher.com

2 http://www.shbiotech.com

3 http://www.roche.com/

4 http://rsb.info.nih.gov/ij/

5 http://www.sas.com/en_us/software/sas9.html

## Supplementary Material

FIGURE S1 Sequencing saturation analysis of physic nut roots and leaves under Pi-deficiency (P-) and Pi-sufficient (P+) conditions after treatment for 2 or 16 days.

FIGURE S2 Results of real-time PCR analysis of changes in expression ratios of the Pi-inducible genes in physic nut leaves and roots after Pi deficiency for 2 and 16 days. The results are based on calculations using two independent loading controls, *JcActin* and H(+)-ATPase, that had steady levels of transcripts in various tissues of physic nut. Since the results of using both loading controls were consistent, results obtained using the *JcActin* gene are presented. Fold changes indicate ratios of expression levels under P- conditions to those recored under P+ conditions at each time point.

FIGURE S3 *JcERF035* over-expressing plants are more sensitive to salt stress. For salt stress tests, 6-day-old wild-type and transgenic Arabidopsis seedlings were transferred to absorbent cotton infiltrated with Hoagland nutrient solution containing 0 mM (mock), or 150 mM NaCl in glass bottles. Ten days after transfer, the seedlings were photographed **(A)** and the fresh weights of their shoots were measured **(B)**.

TABLE S1 Primers used in this study.

TABLE S2 The genes most strongly (≥3-fold) differentially expressed in roots **(A)** and leaves **(B)** under Pi-deficiency 2 and 16 days after the onset of treatment.

TABLE S3 Expression levels of genes (TPM) involved in Pi homeostasis after 2 and 16 days of the P- and P+ treatments.
