# Supplementary figures and images for: Overexpression of a Phosphate Starvation Response AP2/ERF Gene From Physic Nut in Arabidopsis Alters Root Morphological Traits and Phosphate Starvation-Induced Anthocyanin Accumulation
Source: Front Plant Sci. 2018 Aug 20;9:1186. doi: 10.3389/fpls.2018.01186 (PMC6109760; doi:10.3389/fpls.2018.01186)

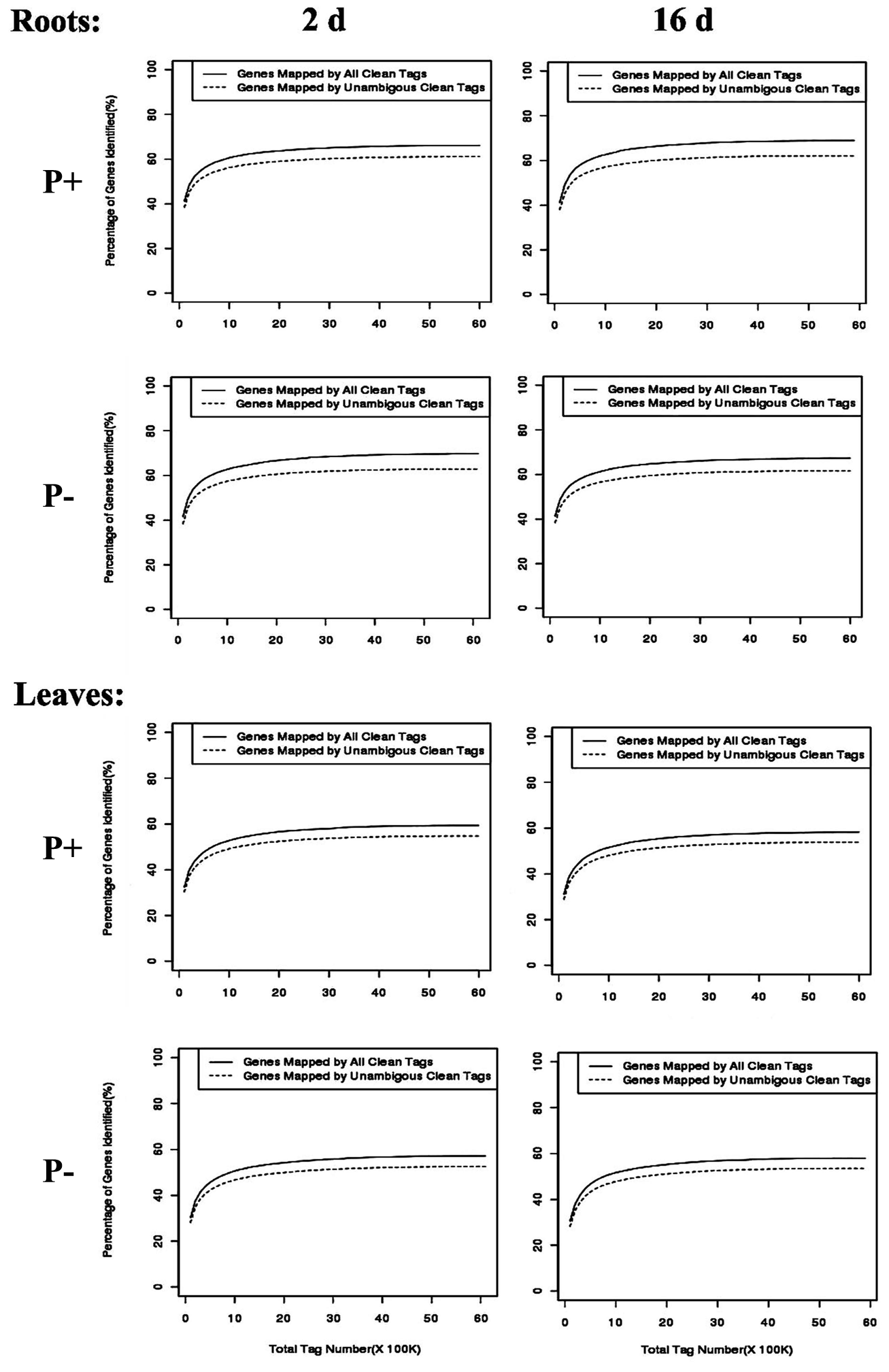

Supplement: FIGURE S1 — Sequencing saturation analysis of physic nut roots and leaves under Pi-deficiency (P-) and Pi-sufficient (P+) conditions after treatment for 2 or 16 days. [file Image_1.TIF]

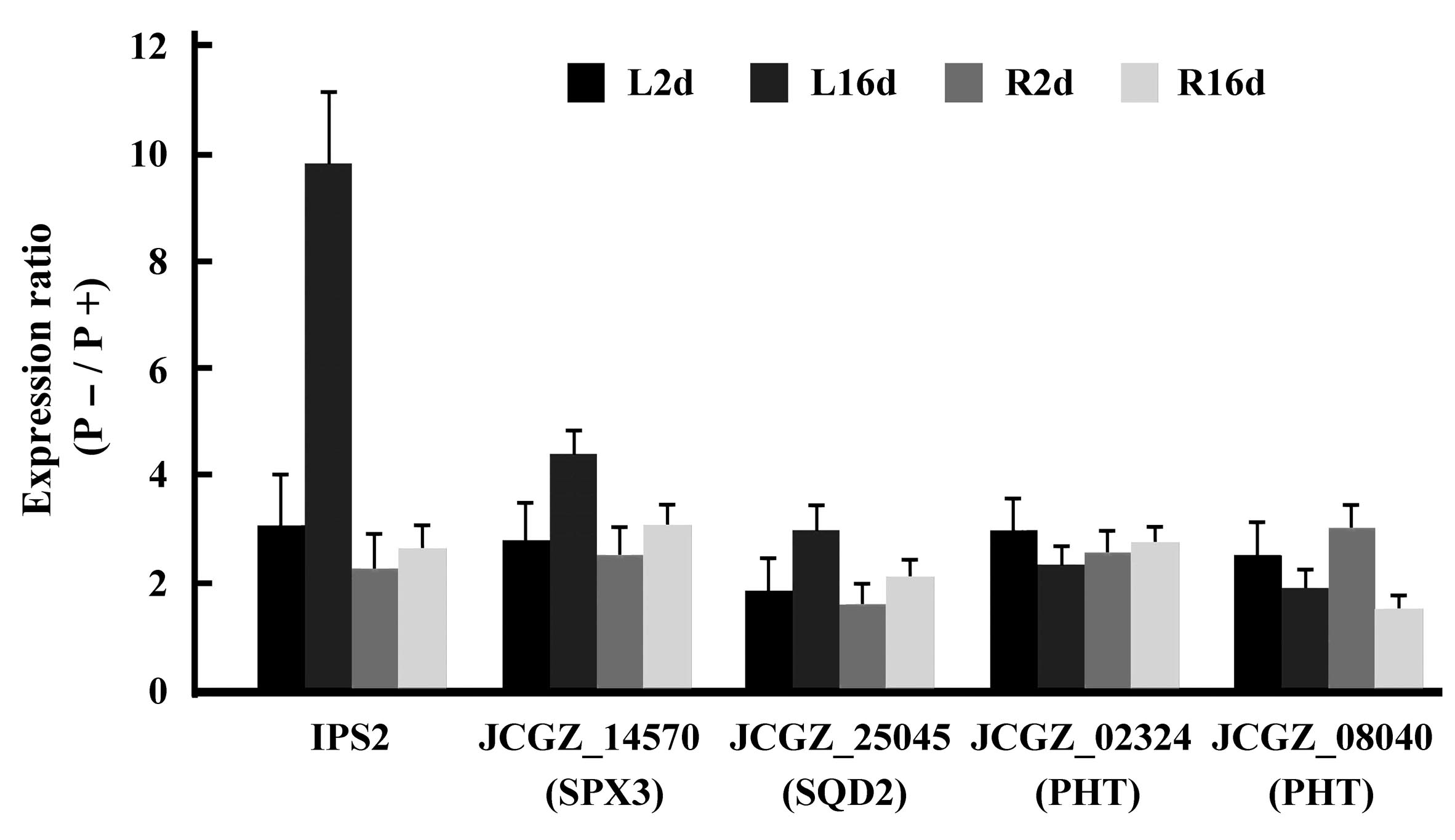

Supplement: FIGURE S2 — Results of real-time PCR analysis of changes in expression ratios of the Pi-inducible genes in physic nut leaves and roots after Pi deficiency for 2 and 16 days. The results are based on calculations using two independent loading controls, JcActin and H(+)-ATPase, that had steady levels of transcripts in various tissues of physic nut. Since the results of using both loading controls were consistent, results obtained using the JcActin gene are presented. Fold changes indicate ratios of expression levels under P- conditions to those recored under P+ conditions at each time point. [file Image_2.JPEG]

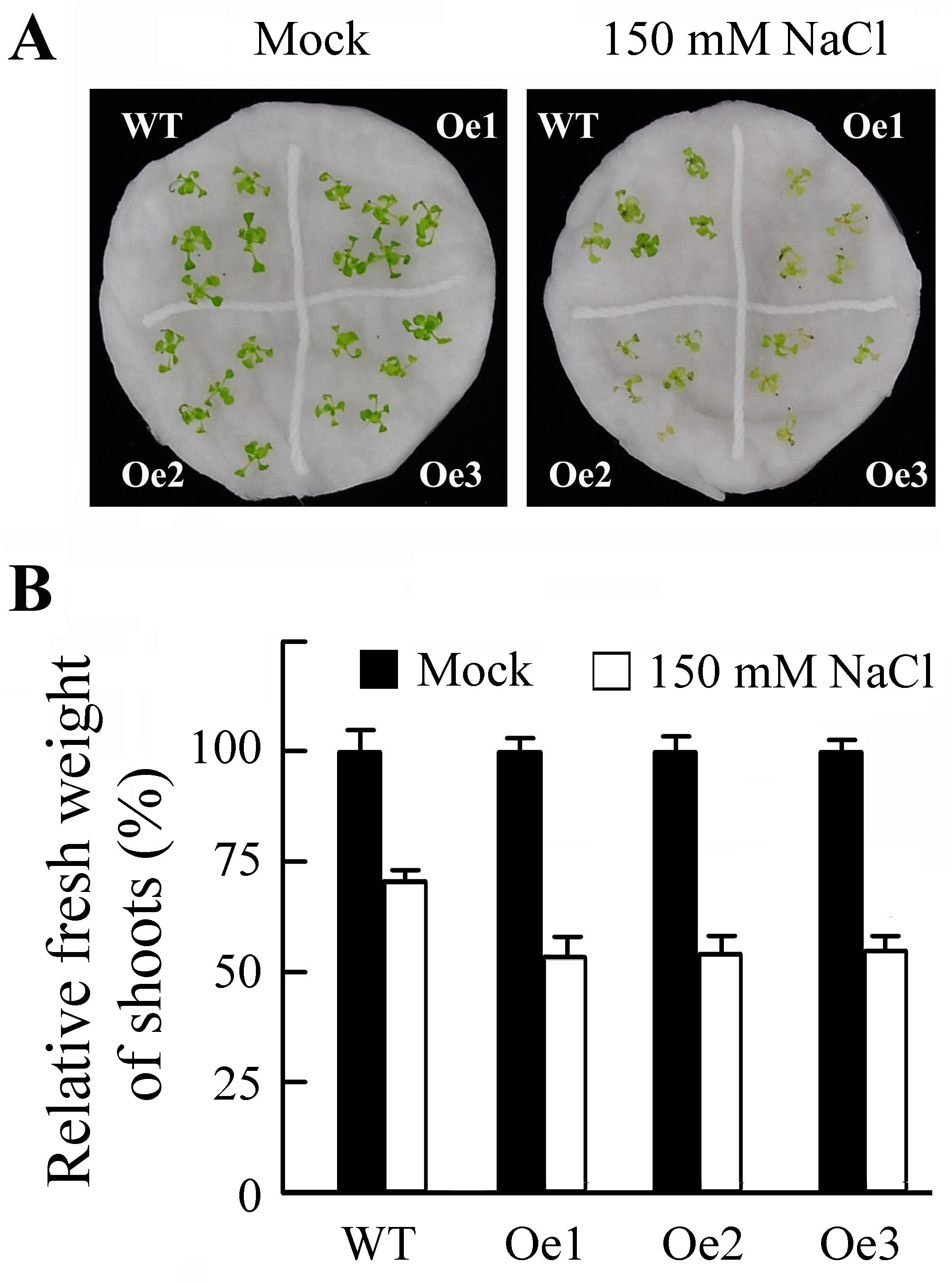

Supplement: FIGURE S3 — JcERF035 over-expressing plants are more sensitive to salt stress. For salt stress tests, 6-day-old wild-type and transgenic Arabidopsis seedlings were transferred to absorbent cotton infiltrated with Hoagland nutrient solution containing 0 mM (mock), or 150 mM NaCl in glass bottles. Ten days after transfer, the seedlings were photographed (A) and the fresh weights of their shoots were measured (B). [file Image_3.JPEG]
